# Membrane Damage during *Listeria monocytogenes* Infection Triggers a Caspase-7 Dependent Cytoprotective Response

**DOI:** 10.1371/journal.ppat.1002628

**Published:** 2012-07-12

**Authors:** Sara K. B. Cassidy, Jon A. Hagar, Thirumala Devi Kanneganti, Luigi Franchi, Gabriel Nuñez, Mary X. D. O'Riordan

**Affiliations:** 1 Department of Microbiology and Immunology, University of Michigan Medical School, Ann Arbor, Michigan, United States of America; 2 Department of Immunology, St. Jude Children's Research Hospital, Memphis, Tennessee, United States of America; 3 Department of Pathology and Comprehensive Cancer Center, University of Michigan Medical School, Ann Arbor, Michigan, United States of America; University of Toronto, Canada

## Abstract

The cysteine protease caspase-7 has an established role in the execution of apoptotic cell death, but recent findings also suggest involvement of caspase-7 during the host response to microbial infection. Caspase-7 can be cleaved by the inflammatory caspase, caspase-1, and has been implicated in processing and activation of microbial virulence factors. Thus, caspase-7 function during microbial infection may be complex, and its role in infection and immunity has yet to be fully elucidated. Here we demonstrate that caspase-7 is cleaved during cytosolic infection with the intracellular bacterial pathogen, *Listeria monocytogenes*. Cleavage of caspase-7 during *L. monocytogenes* infection did not require caspase-1 or key adaptors of the primary pathways of innate immune signaling in this infection, ASC, RIP2 and MyD88. Caspase-7 protected infected macrophages against plasma membrane damage attributable to the bacterial pore-forming toxin Listeriolysin O (LLO). LLO-mediated membrane damage could itself trigger caspase-7 cleavage, independently of infection or overt cell death. We also detected caspase-7 cleavage upon treatment with other bacterial pore-forming toxins, but not in response to detergents. Taken together, our results support a model where cleavage of caspase-7 is a consequence of toxin-mediated membrane damage, a common occurrence during infection. We propose that host activation of caspase-7 in response to pore formation represents an adaptive mechanism by which host cells can protect membrane integrity during infection.

## Author Summary

Macrophages are critical early responders recruited to sites of bacterial infection. Many intracellular bacterial pathogens subvert or bypass macrophage anti-microbial defenses by expression of virulence factors and toxins. The Gram-positive intracellular pathogen, *Listeria monocytogenes*, secretes a pore-forming toxin that can damage host membranes. We found that toxin-mediated damage during bacterial infection triggered activation of a cysteine protease, caspase-7. Activation of this protease was triggered by low levels of toxin alone, suggesting that sublethal toxin-mediated damage is sensed by the host cell. Infected viable macrophages lacking caspase-7 exhibited increased membrane permeability, suggesting that caspase-7 limits membrane damage in infected host cells. This study reveals an unanticipated cytoprotective role for caspase-7 during intracellular infection and provides evidence for a mechanism by which host cells can initiate an adaptive response to survive membrane-damaging toxins, which are commonly associated with pathogenic microbes.

## Introduction

Pore-forming toxins are integral to the virulence of many microbial pathogens, including the Gram-positive bacterium, *Listeria monocytogenes*. This facultative intracellular pathogen can cause life-threatening disease in humans, particularly in the very old and very young, the immunocompromised, and pregnant women [1]. In macrophages, *L. monocytogenes* gains access to its replicative niche via the action of a pore-forming cholesterol-dependent cytolysin, Listeriolysin O (LLO) [2]. LLO-dependent perforation of the primary phagosomal membrane allows the pathogen to escape into the cytosol, where it grows to high titers in the apparent absence of overt cell damage until late in infection [3], [4]. Virulence of *L. monocytogenes* therefore requires a delicate balance between expressing virulence factors, such as LLO, to survive host cell defenses while maintaining an intact host cell niche. Infection with *L. monocytogenes* expressing an overactive allele of LLO [5], [6] or with a strain that overproduces LLO [7] results in host cell damage and attenuation *in vivo*, primarily due to killing of extracellular *L. monocytogenes* by neutrophils [5]. It can therefore be inferred that the integrity and survival of infected host cells affects virulence of *L. monocytogenes*.

The infected macrophage plays a dichotomous role during *L. monocytogenes* infection, acting both as a reservoir for bacterial replication and as a source for inflammatory signals that result from recognition of microbial ligands or cellular stress. *L. monocytogenes* activates many inflammatory pathways in the cell that promote eventual bacterial clearance and immunity. Infection stimulates Toll-like receptors TLR2 and possibly TLR5, and the Nod-like receptors (NLRs) Nod1 and Nod2, resulting in NF-κB-dependent pro-inflammatory gene transcription [8]–[12]. Cytosolic *L. monocytogenes* triggers assembly of the caspase-1 associated inflammasome, a multiprotein complex whose formation can lead to an inflammatory cell death termed pyroptosis [13]. Active caspase-1 processes pro-IL-1β and pro-IL-18, inflammatory cytokines that promote antimicrobial properties of phagocytes and stimulate adaptive immunity [14]–[16]. Several NLRs activate caspase-1 as a result of *L. monocytogenes* infection, including NLRC4, NLRP3 and AIM2, all of which require the adaptor protein ASC [17]–[20]. Studies in knockout mice have demonstrated that caspase-1 is important for primary clearance of *L. monocytogenes*, but the role of other caspases in the innate immune response to infection is less well defined [21].

Caspase-7 is a member of a family of cytosolic cysteine proteases that promulgate diverse biological responses, including programmed cell death and inflammation. Caspases can also promote cell survival, as caspase-1 positively regulates cholesterol biosynthesis in response to a bacterial pore-forming toxin, aerolysin [22]. A defining characteristic of caspases is specific cleavage of substrates at aspartic acid residues using a cysteine side chain as a nucleophile for peptide hydrolysis [23]. Caspases reside in the cytosol as zymogens that require dimerization and/or proteolytic cleavage before becoming catalytically active. Cleavage of caspase-7 results in a large and a small fragment; proteolysis between the large and small subunit is considered the fundamental activating event [24]. Caspase-7 was initially characterized as an “executioner” caspase whose activity directs the highly regulated cascade of events leading to cellular disassembly during apoptotic cell death ([25], [26] and recently reviewed in [27]). Recent studies have implicated maturation of caspase-7 as a consequence of infection or inflammatory stimulation. Caspase-7 cleavage occurs during infection by the Gram-negative intracellular bacterial pathogens *Salmonella enterica* serovar Typhimurium and *Legionella pneumophila* [28], [29]. In these contexts, caspase-7 was cleaved by inflammasome-associated caspase-1. Caspase-7-deficient macrophages allowed increased *L. pneumophila* intracellular growth, possibly due to delayed macrophage cell death [28]. These studies provide evidence that caspase-7 is involved in host-pathogen interactions.

Here we show that caspase-7 cleavage is triggered by membrane damage during *L. monocytogenes* infection, and is dissociated from canonical markers of apoptosis. Caspase-7 cleavage occurred in the absence of caspase-1, distinct from the activation cascade observed during infection by *S.* Typhimurium and *L. pneumophila* [28], [29]. Infected macrophages lacking caspase-7 exhibited increased plasma membrane permeability, which required ongoing production of LLO. Treatment of host cells with sublytic concentrations of recombinant LLO, as well as a pore-forming toxin from *Staphylococus aureus*, α-hemolysin, triggered caspase-7 activation in the absence of infection. Together, these data lead us to propose that caspase-7 activation is a protective host response to plasma membrane damage that limits subsequent cytotoxicity during bacterial infection.

## Results

### Caspase-7 cleavage is induced by *L. monocytogenes* infection without concomitant induction of apoptosis

**Figure 1:**
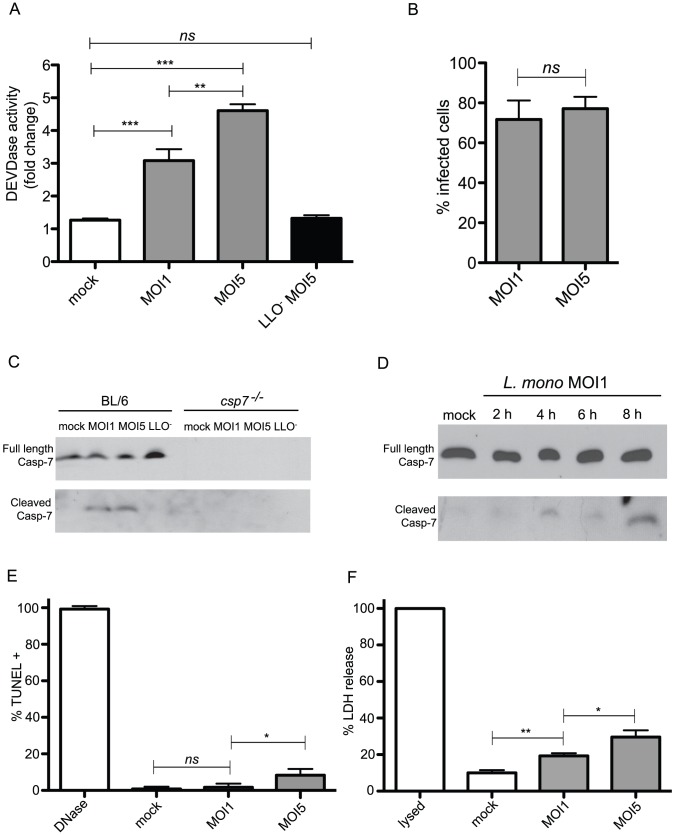
Caspase-7 is activated in macrophages during infection with *L. monocytogenes* with minimal evidence of apoptosis. (A) BL/6 BMDM were infected or not (mock) at the indicated multiplicities of infection with either WT *L. monocytogenes* or with a mutant strain lacking the *hly* gene encoding Listeriolysin O (LLO^−^). For this and all future experiments, BMDM infected with the LLO^−^ strain were infected at MOI5. Cells were assessed for enzymatic activity 8 hours post infection. (B) The percentage of cells infected with *L. monocytogenes* at 8 h pi at the given MOI was assessed using fluorescence microscopy and Metamorph software. (C) BL/6 and caspase-7-deficient macrophages were infected with *L. monocytogenes* and cell lysates were probed for the cleavage of caspase-7 by immunoblot using an antibody that recognizes both full length and cleaved caspase-7 protein. The paired panels for full length and cleaved caspase-7 shown in this and all other immunoblots are from the same blot, but were cut to allow for longer exposure of the cleaved fragment. (D) BL/6 macrophages were infected with *L. monocytogenes*, lysates were harvested at the indicated times post infection and probed for caspase-7 protein. (E) BL/6 macrophages were infected and assessed for TUNEL staining as a marker of apoptosis 8 h pi. DNAse treatment was used as a positive control for DNA fragmentation. P values were generated using unpaired t-tests of no less than 300 cells per condition. (F) Lactate dehydrogenase (LDH) release into cell culture supernatants was measured 8 h pi as a marker of cell integrity. The amount of LDH released as the result of detergent induced lysis was set to 100% and the signal from untreated cells was set as baseline. (A–F) are representative of no less than three independent experiments. * P<0.05, ** P<0.01 and *** P<0.001, generated using unpaired t-tests. *ns* = not significant.

We first investigated whether hallmarks of proteolysis associated with caspase-7 activity could be detected in *L. monocytogenes* infected cells. To this end, we infected bone marrow derived macrophages (BMDM) from C57BL/6 (BL/6) mice with either a WT strain of *L. monocytogenes*, or with a strain lacking the *hly* gene, which encodes the pore-forming toxin Listeriolysin O (LLO^−^ strain). The LLO^−^ strain cannot escape the primary phagosome and does not replicate within macrophages. At 8 h pi, we assessed DEVDase activity, an indicator of caspase-3/7 proteolytic activity, by measuring cleavage of a luminescent DEVD containing substrate in cell lysates (Figure 1A). Both caspase-3 and -7 are able to cleave exogenous DEVD substrate, but recent studies suggest that proteases have overlapping but distinct physiological substrates within the host cell [30]. We detected an increase in DEVD-specific enzymatic activity in response to infection with WT, but not the LLO^−^ mutant, indicating that bacterial uptake per se was insufficient to stimulate DEVDase activity. The difference in DEVDase activity between cells infected at MOI1 vs. MOI5 was not attributable to differences in the number of infected cells (Figure 1B) nor the number of bacteria, as we isolated nearly equivalent CFU from cultures infected under these conditions at 8 h pi (Figure S1). At MOI1 and MOI5, 80–90% of cells were infected, although at MOI5, macrophages contained higher numbers of bacteria at early time points. To specifically determine if caspase-7 was activated during *L. monocytogenes* infection, we investigated whether *L. monocytogenes* infection induced caspase-7 cleavage in BMDM by lysing infected cells at 8 h pi and analyzing caspase-7 by immunoblot using an antibody that recognizes both the full length protein and the larger cleavage product (Figure 1C). We observed cleaved caspase-7 protein in BL/6 cells infected with WT at MOI1 and MOI5, but not in uninfected (mock) BMDM or BMDM infected with the LLO^−^ strain (MOI 5), suggesting bacterial occupancy of the phagosome was insufficient to stimulate cleavage of this protease. Full-length caspase-7 protein was detected in BL/6 BMDM in all samples and was used as a protein loading control since the overall abundance of the cleaved caspase-7 product was low compared to full length. We also assessed caspase-3 cleavage during *L. monocytogenes* infection by immunoblot (Figure S2). Although we detected cleavage of caspase-3 in response to infection, the cleavage of caspase-7 in response to infection was more robust, and we therefore decided to further investigate caspase-7. To determine the kinetics of caspase-7 activation during infection, we assayed cell lysates for cleavage at 2, 4, 6 and 8 hpi (Figure 1D). Cleavage of caspase-7 was detected between 4 and 8 hpi, by which time the majority of *L. monocytogenes* are replicating in the cytosol. Taken together, these data indicate that caspase-7 is activated during *L. monocytogenes* infection, and requires LLO.

Caspase-7 was originally identified as an executioner caspase whose activity was associated with activation of the apoptotic cascade [31]. To determine whether caspase-7 cleavage was also associated with molecular markers of programmed cell death during *L. monocytogenes* infection, we first quantified the number of infected cells at 8 hpi by staining samples with anti-*Listeria* antibody, followed by immunofluorescence microscopy (Figure 1B). We then prepared infected cell cultures for TUNEL staining to visualize DNA fragmentation. BMDM were exposed to *L. monocytogenes* at MOI1 or MOI5, and at 8 h pi cells were fixed and analyzed for DNA fragmentation by immunofluorescence microscopy. In agreement with previous findings [3], we found no significant increase in DNA fragmentation in cells infected at MOI1 compared to uninfected control cells (Figure 1E). At MOI5, there was a statistically significant increase in the number of TUNEL positive cells compared to uninfected cells. However, comparing the overall number of TUNEL positive cells even in the MOI5 infection (∼10–15%) to the number of cells infected (80–90%), we conclude that the majority of cells infected with *L. monocytogenes* do not display this characteristic hallmark of apoptosis by 8 h pi. We obtained similar results using Annexin V to measure phosphatidylserine exposure, an early event in apoptotic cell death (Figure S3). We also measured lactate dehydrogenase (LDH) release at 8 h pi as an indicator of overall cell viability (Figure 1F). *L. monocytogenes* infection induced a modest but notable increase in LDH release at 8 h pi compared to uninfected cells. This range is consistent with previously published reports [3], and indicates that at low MOI the majority of cells infected with *L. monocytogenes* retain LDH. We conclude from these results that *L. monocytogenes* infection stimulates caspase-7 cleavage, but is not predominantly associated with molecular markers of apoptotic cell death.

### Key regulators of innate immune signaling are dispensable for caspase-7 activation during *L. monocytogenes* infection

**Figure 2:**
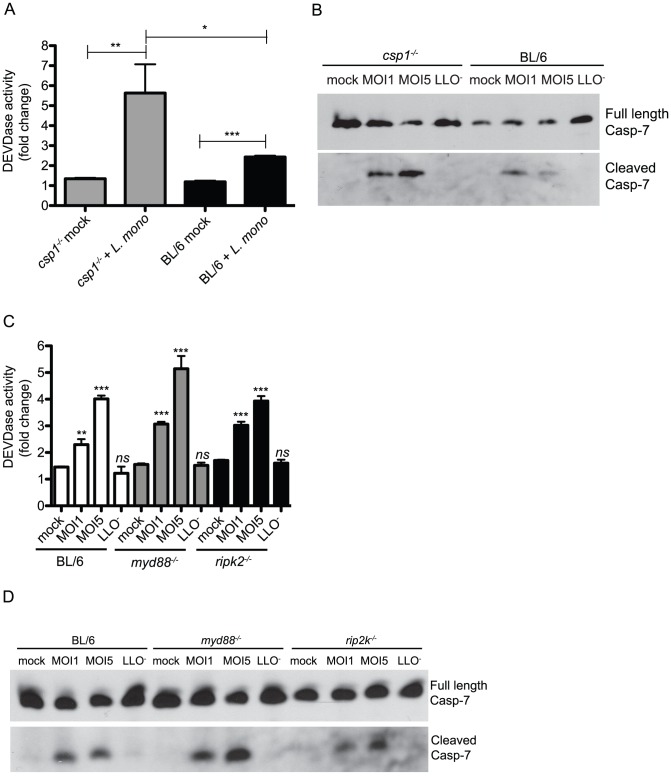
Caspase-1 and other key mediators of innate immunity are individually dispensable for caspase-7 cleavage. (A) Caspase-1 deficient and BL/6 BMDM were infected as indicated and lysed at 8 h pi. DEVDase activity in the lysates was measured by Caspase-Glo 3/7 assay. Signal from assay buffer alone was subtracted from all the sample values. (B) BMDM were infected as in (A) and cells were lysed at 8 h pi. Lysates were subjected to SDS-PAGE and immunoblotting with an anti-caspase-7 antibody that recognizes both full length and cleaved caspase-7 protein. (C) BL/6, MyD88- deficient and RIP2-deficient BMDM were infected as indicated and lysed at 8 h pi. DEVDase activity in the lysates was measured by Caspase-Glo 3/7 assay. Signal from assay buffer alone was subtracted from all the sample values. (D) BMDM were infected as in (A) and cells were lysed at 8 h pi. Lysates were subjected to SDS-PAGE and immunoblotting with the anti-caspase-7 antibody. Statistical significance was determined by Student's unpaired t-test; *p<0.05, **p<0.01 and ***p<0.001.

*L. monocytogenes* infection triggers activation of caspase-1, and during some bacterial infections, caspase-7 can be a substrate of caspase-1 [28], [29]. We therefore tested whether the activation of caspase-7 during *L. monocytogenes* infection was dependent on the presence of caspase-1 by infecting *csp1^−/−^* BMDM and measuring DEVDase activity. We detected increased DEVDase activity upon infection of caspase-1 deficient BMDM (Figure 2A). Analysis of caspase-7 protein by immunoblot of infected cell lysates definitively revealed cleavage of caspase-7 in *csp1^−/−^* BMDM upon infection, and this cleavage was dependent on LLO (Figure 2B). ASC is a key adaptor protein necessary for caspase-1 activation during inflammasome formation [32]. To determine if ASC was necessary for caspase-7 cleavage, we assayed caspase-7 cleavage in infected *Asc^−/−^* BMDM and found that caspase-7 activation also did not require ASC during *L. monocytogenes* infection (data not shown). These data demonstrate that the mechanism of caspase-7 cleavage during *L. monocytogenes* infection does not require caspase-1, and thus appears to be distinct from mechanisms reported for other intracellular bacterial pathogens.

Extracellular (TLR2 and TLR5) and intracellular (Nod1 and Nod2) pattern recognition receptors are able to sense *L. monocytogenes* and direct transcription of cytokines and chemokines to promote inflammation and clearance [8]–[12]. To determine whether these bacterial recognition pathways contributed to caspase-7 activation upon infection, we evaluated *L. monocytogenes*-induced DEVDase activity and caspase-7 cleavage in BMDM from *myd88^−/−^* and *rip2k^−/−^* mice. MyD88 is a critical adaptor that mediates signaling for 9 of the 10 TLRs with known ligands. RIP2 is a protein kinase that mediates inflammatory signaling through Nod1 and Nod2. We observed infection-induced DEVDase activity and caspase-7 cleavage in *myd88^−/−^* and *rip2k^−/−^* BMDM at levels comparable to WT BMDM (Figure 2C and 2D). We also detected caspase-7 cleavage in *nod1^−/−^* and *nod2^−/−^* macrophages comparable to WT (data not shown). From these results, we conclude that the innate immune signaling pathways regulated by MyD88-dependent TLRs, Nod1, Nod2, ASC, and caspase-1 are individually dispensable for caspase-7 activation during *L. monocytogenes* infection.

### Caspase-7 deficient BMDM are permeable to small molecules during infection

**Figure 3:**
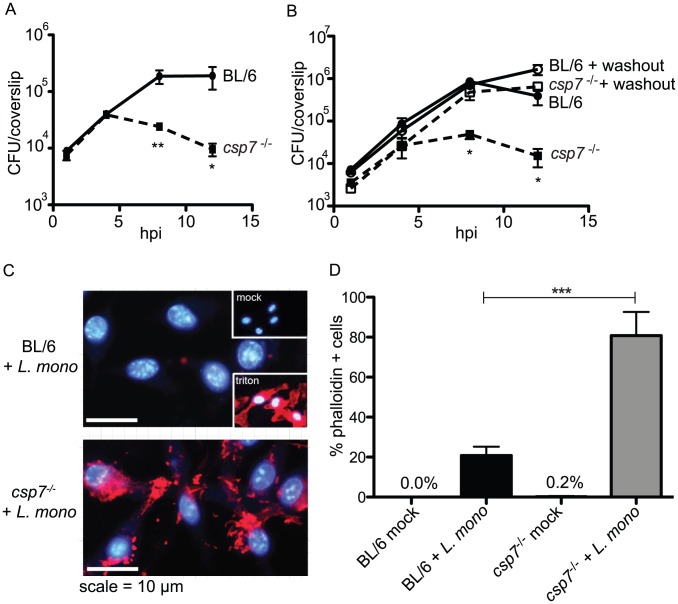
Caspase-7 deficient BMDM are permeable to small molecules during infection. (A) BMDM were infected with *L. monocytogenes* and gentamicin (480 Da) was added 30 min pi to abrogate extracellular bacterial growth. (B) Cells were infected as in (A), but beginning at 2 h pi, cells were washed with PBS and replenished with antibiotic free medium once an hour to limit extracellular bacterial growth. (C) BL/6 and *csp7^−/−^* cells infected for 8 h with *L. monocytogenes* were incubated with rhodamine-phalloidin (∼1200 Da) for 15 min prior to fixation and DAPI counterstain. The insets show rhodamine-phalloidin staining of mock treated and Triton X-100 treated BL/6 macrophages. Samples were visualized by epifluorescence microscopy. (D) Cells were treated as in (C) and phalloidin influx was quantified by fluorescence microscopy (300 cells were counted per condition per experiment). Error bars represent standard deviation from the mean. All conditions were measured in triplicate, and all experiments were repeated ≥3 times. Statistics were calculated using the Student's unpaired t-test; *p<0.05, **p<0.01 and ***p<0.001.

Since caspase-7 was cleaved during infection with *L. monocytogenes*, we hypothesized that the activity of this enzyme could impact intracellular infection. We therefore measured *L. monocytogenes* replication in BL/6 and *csp7^−/−^* BMDM in an antibiotic protection assay where 50 µg/ml of the cell impermeant antibiotic gentamicin was added 30 min pi to eliminate extracellular bacteria (Figure 3A). We observed significant decline in the number of intracellular bacteria over time within the *csp7^−/−^* BMDM compared to BL/6 cells. We considered the possibility that *csp7^−/−^* cells were becoming permeable to gentamicin, allowing antibiotic influx into the intracellular space, killing intracellular bacteria. To test this idea, we performed a gentamicin washout, removing gentamicin from the medium 2 h pi, after which antibiotic-free medium was added and replaced every hour to limit extracellular bacterial growth (Figure 3B). When gentamicin was removed from the extracellular space, there was no significant difference in bacterial replication between BL/6 and *csp7^−/−^* macrophages. These data suggest increased plasma membrane permeability of *L. monocytogenes-*infected macrophages in the absence of caspase-7.

To more directly assess membrane integrity of *L. monocytogenes* infected BMDM, we evaluated their permeability to small fluorescent molecules during infection. At 8 h pi we exposed live infected cells to rhodamine-phalloidin (Rh-P), a small (1200 Da) cell impermeant compound that binds F-actin in the host cell. After a 15-minute exposure, the cells were fixed and counter-stained with DAPI. Permeability to Rh-P was compared between *csp7^−/−^* and BL/6 BMDM using epifluorescence microscopy (Figure 3C and 3D). Untreated BMDM excluded Rh-P, while cells treated with Triton X-100 were fully permeable to Rh-P (Figure 3C insets). Although Rh-P permeability was similar between genotypes in the absence of infection, during infection the overall number of cells stained with phalloidin was significantly greater in the *csp7^−/−^* macrophages compared to wildtype BMDM. Notably, despite permeability to small molecules, infected *csp7^−/−^* cells displayed no significant increase in LDH release at 8 h pi compared to control cells (Figure S4). These findings suggest that membrane damage occurring during *L. monocytogenes* infection may be transient, and is insufficient to allow efflux of the large LDH tetramer (≈137,000 Da) [33]. Taken together, these data indicate that caspase-7 promotes plasma membrane integrity during infection with *L. monocytogenes*.

### Plasma membrane instability during *L. monocytogenes* infection requires LLO

**Figure 4:**
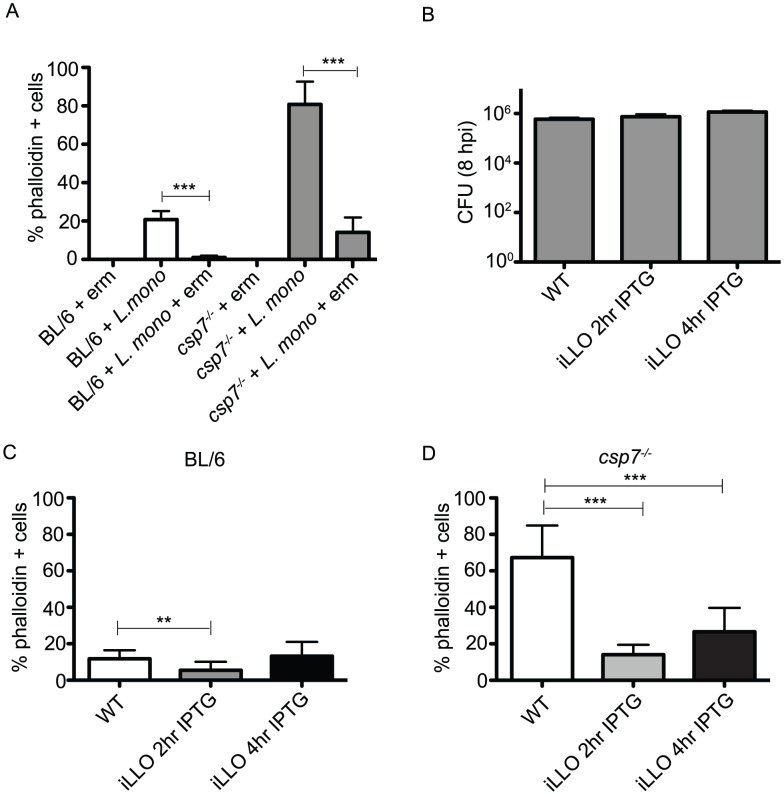
Caspase-7 enhances host resistance to LLO-mediated plasma membrane damage. (A) Caspase-7-deficient and BL/6 BMDM were infected with *L. monocytogenes*. At 4 h pi, 10 µg/ml erythromycin (erm) was added to the medium to inhibit bacterial protein synthesis, and was maintained for the duration of the experiment. At 8 h pi, the cells were incubated with rhodamine-phalloidin and visualized by fluorescence microscopy as in Figure 3CD to measure permeability. (B) BMDM were infected with WT *L. monocytogenes* or a bacterial strain expressing an inducible allele of LLO (iLLO). BMDM infected with the iLLO strain were incubated with 1 mM IPTG for 2 h or 4 h as indicated. Intracellular colony forming units were measured at 8 h pi. (C) BL/6 BMDM were infected as in (B), and rhodamine-phalloidin permeability was quantified at 8 h pi by epifluorescence microscopy. (D) Caspase-7-deficient BMDM were infected as in (B), and rhodamine-phalloidin permeability was quantified at 8 h pi by epifluorescence microscopy. Statistics were calculated using the Student's unpaired t-test; **p<0.01 and ***p<0.001.

Plasma membrane damage during *L. monocytogenes* infection could be the result of toxin-mediated pore formation. To determine whether bacterial protein synthesis was needed to induce host membrane instability, we treated infected cells with a bacteriostatic concentration of erythyromycin (erm), a macrolide antibiotic that targets the bacterial 50S ribosomal subunit. Erythromycin was added to infected cells at 2 h pi, and at 8 h pi we found a significant reduction in the number of caspase-7-deficient phalloidin positive cells upon inhibition of bacterial protein synthesis (Figure 4A). We then tested the requirement for LLO in driving the plasma membrane damage we observed in infected caspase-7 deficient macrophages. LLO is an oligomeric cytolysin that forms pores in cholesterol-containing membranes [34]. The oligomerization of LLO occurs optimally at acidic pH [6], [35] but still maintains some pore-forming activity at neutral pH [36]–[38]. LLO is actively produced by cytosolic *L. monocytogenes* [39]. We hypothesized that LLO production by cytosolic bacteria could result in continual but transient membrane damage during infection, which was exacerbated in *csp7^−/−^* cells. To test this hypothesis, we used a strain of *L. monocytogenes* with LLO expression controlled by an IPTG inducible promoter (iLLO) [40]. We transiently induced production of LLO during infection by the iLLO-expressing strain to permit escape from the phagosome, and then removed IPTG from the cell culture medium. Determination of endpoint CFU at 8 h pi in BL/6 macrophages infected with the WT and iLLO strains revealed no significant differences in the number of intracellular bacteria between strains (Figure 4B). However, IPTG removal at 2 h pi resulted in significantly less phalloidin permeable BL/6 BMDM infected with the iLLO strain compared to cells infected with WT bacteria at 8 h pi (Figure 4C). The reduction of permeability in *csp7^−/−^* macrophages infected with the iLLO-expressing strain compared to the WT strain was even more pronounced, supporting a protective role for this protease against infection associated membrane damage (Figure 4D). Increasing induction time from 2 to 4 hours increased the number of phalloidin positive cells in both genotypes. Therefore, we conclude that LLO is required to drive the membrane permeability defect in macrophages lacking caspase-7.

### Pore formation by exogenous toxin is a trigger for caspase-7 cleavage

**Figure 5:**
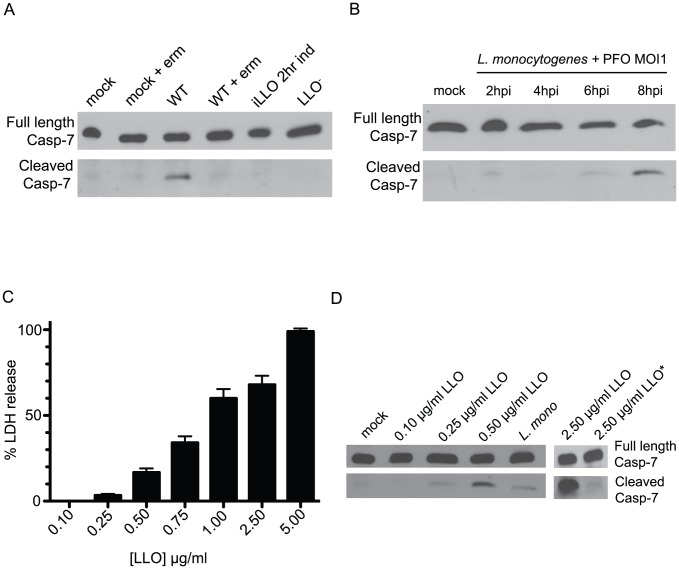
Caspase-7 is cleaved in response to membrane damage in the absence of infection. (A) BL/6 BMDM were infected with the indicated strains and lysed at 8 h pi. Lysates from uninfected (mock), mock infected cells treated with erythromycin (erm), WT infected, WT infected+erm 2 h pi, iLLO infected+IPTG for 2 h pi, and LLO^−^ infected were subjected to SDS-PAGE and immunoblot analysis with an anti-caspase-7 antibody that recognizes both the full length and cleaved protein. (B) BL/6 BMDMs were infected with a strain of *L. monocytogenes* expressing PFO in place of LLO and cell lysates were harvested at the given times post infection. Lysates were probed for caspase-7 protein using the anti-caspase-7 antibody. (C) BL/6 macrophages were incubated with the indicated concentrations of recombinant LLO protein for 1 h after which the media was assayed for lactate dehydrogenase activity. The amount of LDH release from detergent lysed cells was set to 100% and the LDH released from untreated cells was set to 0%. (D) BMDMs treated as in (C) were lysed at 1 h post treatment and assayed for caspase-7 cleavage by SDS-PAGE and immunoblot. The LLO* sample was heat treated for 10 minutes at 65°C before addition to BMDMs to inactivate the toxin.

Bacterial production of LLO was required for permeability of caspase-7 deficient macrophages during infection (Figure 4), leading us to question whether LLO was also the trigger that stimulated cleavage of caspase-7. To test this possibility, we infected BMDM with *L monocytogenes*, and then added 10 µg/ml erm at 2 h pi to inhibit bacterial protein synthesis and assayed for caspase-7 cleavage by immunoblot. We were unable to detect cleavage of caspase-7 when erm was added to infected cell cultures, demonstrating that bacterial protein synthesis was necessary to stimulate this response (Figure 5A). We also infected BMDM with the IPTG-inducible LLO expressing strain, incubating with IPTG for 2 h pi to promote vacuolar escape, followed by removal of IPTG from the medium to limit LLO production. Although the iLLO and WT strains grew to equivalent intracellular CFU under these conditions (Figure S5), we only observed caspase-7 cleavage in cells infected with the WT strain, not the iLLO strain (Figure 5A). LLO is one of a large family of cholesterol dependent cytolysins (CDC) produced by Gram-positive bacterial pathogens. To determine whether activation of caspase-7 during bacterial infection was specific to LLO, or whether other CDC toxins behaved similarly, we infected wildtype and caspase-7^−/−^ BMDM with *L. monocytogenes* expressing the related CDC toxin perfringolysin O (PFO) instead of LLO [41]. *L. monocytogenes* expressing PFO also stimulated caspase-7 activation ( 5B). Thus, we find that cytosolic bacterial replication per se is insufficient to stimulate caspase-7 cleavage, and that ongoing production of toxin is necessary for activation of the protease during *L. monocytogenes* infection.

We next asked if exogenous LLO could activate caspase-7 independent of infection. To this end, we first determined the sublytic concentration range of purified recombinant LLO (rLLO), using LDH release as a marker for loss of viability at 1 h post treatment (Figure 5B). We then probed cell lysates incubated for 1 h with rLLO for caspase-7 cleavage by immunoblot. Concentrations of exogenous rLLO, i.e., 0.25 µg/ml, that only induced a low level of LDH release (∼5%) from BMDM up to 5 h pi (Figure 5C and Figure S6) were sufficient to stimulate caspase-7 cleavage (Figure 5D). As we increased the concentration of exogenous LLO, we saw a concomitant increase in LDH release that correlated with more robust caspase-7 cleavage (Figure 5C and 5D, and data not shown). These data demonstrate that sublytic concentrations of LLO can activate caspase-7 in the absence of infection. To address whether LLO in its native conformation was necessary to stimulate caspase-7 cleavage, we heat-treated the protein for 10 minutes at 65°C (LLO*), and compared caspase-7 cleavage against cells intoxicated with the same concentration of native protein. When LLO was heat-inactivated, we observed significantly less caspase-7 cleavage compared to cells treated with active toxin (Figure 5D). These data show that native LLO is necessary for induction of caspase-7 cleavage. One interpretation of these data could be that a native epitope of LLO stimulates caspase-7 cleavage through binding of a host receptor at the plasma membrane. However, since we observed LLO-dependent caspase-7 cleavage whether LLO was extracellular or in the cytosol, we propose instead that LLO triggers caspase-7 cleavage through pore formation.

### Pore-forming toxins, but not detergents, stimulate caspase-7 cleavage

**Figure 6:**
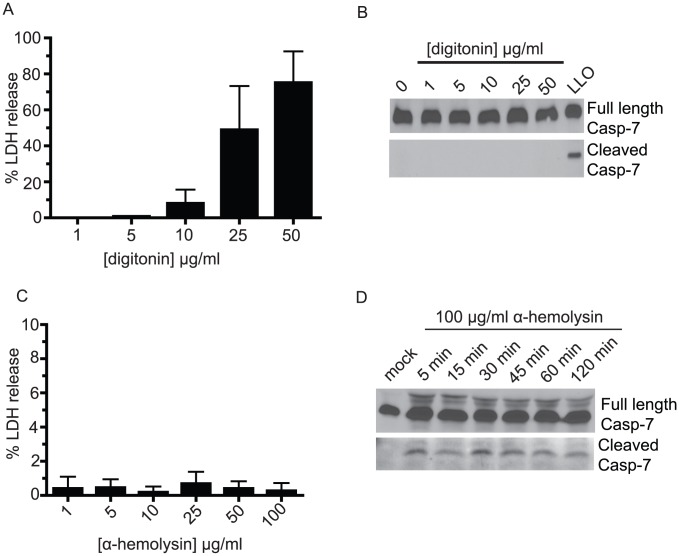
Damage caused by pore-forming toxins stimulates caspase-7 cleavage. (A) BMDMs were treated with digitonin for 1 h after which the cell media was tested for lactate dehydrogenase activity. (B) BMDM treated as in (A) were lysed 1 h post treatment and assayed for caspase-7 cleavage by SDS-PAGE and immunoblot using an antibody that recognizes both the full length and cleaved protein. Treatment of BMDM with 0.75 µg/ml recombinant LLO was included as a control for caspase-7 cleavage. (C) BL/6 BMDM were treated with varying concentrations of α-hemolysin (Sigma-Aldrich) and cell media was tested for lactate dehydrogenase activity 1 h post treatment. The amount of LDH release from detergent lysed cells was set to 100% and the LDH released from untreated cells was set to 0%. (D) BMDM were treated with 100 µg/ml α-hemolysin and were lysed at the given times post treatment. Lysates were probed for caspase-7 cleavage by immunoblot.

To address whether caspase-7 cleavage occurs as a general response to membrane damage, we assessed the ability of exogenous detergent treatment to activate the protease. Digitonin is a non-ionic glycoside detergent that can reversibly permeabilize the plasma membrane by forming complexes with cholesterol [42]. To determine if caspase-7 could be activated in response to detergent permeabilization, we treated BMDM with lytic and sublytic concentrations of digitonin and probed for caspase-7 cleavage 1 h post treatment by immunoblot. Although digitonin treatment induced plasma membrane damage, as assessed by measuring LDH release (Figure 6A), detergent treatment did not stimulate caspase-7 cleavage at any concentration tested (Figure 6B). We obtained similar results using a sublytic to lytic range of the detergents Nonidet P-40, Triton-X100, Tween-20 and saponin (data not shown). These data indicate that plasma membrane damage alone is insufficient to stimulate caspase-7 activation.

We then asked if caspase-7 cleavage could be stimulated by other pore-forming toxins (PFT). *Staphylococcus aureus* α-hemolysin is a well studied PFT distinct from the CDC family of toxins. Treatment of BMDM with α-hemolysin resulted in little to no LDH release up to 8 h post treatment (Figure 6C and S7), consistent with previous reports that nucleated cells can repair damage caused by this toxin [43]–[45]. To test the pore-forming ability of the toxin we confirmed it was lytic on red blood cells (data not shown). α-hemolysin did trigger caspase-7 activation as early as 5 min post treatment (Figure 6D). Some cells types activate the apoptotic cascade in response to treatment with low doses, but not high doses, of α-hemolysin [46], [47]. However we observed no morphological hallmarks of apoptosis up to 8 h post toxin treatment (data not shown). These results suggest that caspase-7 activation is generally responsive to membrane damage by bacterial pore-forming toxins.

## Discussion

**Figure 7:**
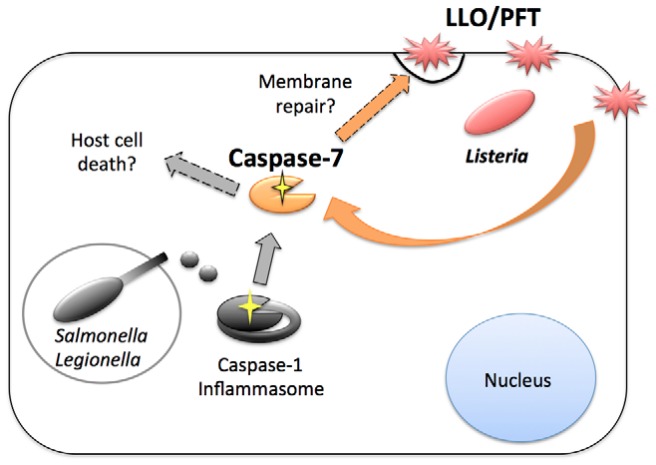
Model for caspase-7 activation during bacterial infection. The bacterial pathogens *L. pneumophila* and *S.* Typhimurium secrete proteins such as flagellin into the host cell during infection, triggering the inflammasome and activation of caspase-1. Caspase-1 can then direct the cleavage of caspase-7 [28], [29]. During *L. pneumophila* infection, caspase-7 activation may promote infected cell death [28] (grey arrows). During *L. monocytogenes* infection (orange arrows) caspase-7 is activated independently from caspase-1 and other innate immune signaling mediators. Instead, caspase-7 cleavage is triggered by membrane damage and can occur in the absence of infection when cells are treated exogenously with pore-forming toxins. In this context, caspase-7 activity promotes the integrity of the host cell membrane.

Here we report that caspase-7 protects plasma membrane integrity during infection with *L. monocytogenes*. We found that the transient membrane damage observed during infection was dependent on the pore-forming toxin, LLO. We also showed that caspase-7 cleavage by *L. monocytogenes* infection did not individually require caspase-1, ASC, MyD88-dependent TLR, or Nod1 and Nod2 signaling. Instead, we found that sublytic membrane damage by recombinant LLO and α-hemolysin, even in the absence of infection, could stimulate caspase-7 activation. *L. monocytogenes* expressing PFO also stimulated caspase-7 activity. However, treatment of BMDM with sublytic concentrations of detergent did not trigger caspase-7 cleavage. These data suggest that general membrane damage or changes in ion gradients across the membrane are insufficient to stimulate caspase-7 and the subsequent cytoprotective response. Although these observations do not rule out a role for pattern recognition of specific microbial ligands in activation of caspase-7, our results suggest the possibility that caspase-7 participates in sensing and/or repair of PFT-induced membrane damage in the infected cell by a caspase-1 independent mechanism (Figure 7). Given the abundance and importance of PFTs for many pathogens, we propose a model whereby caspase-7 is induced by plasma membrane damage that occurs during microbial infection and initiates mechanisms of phagocyte membrane repair to protect the infected cell.

Caspases are well studied for their pivotal roles in apoptotic and inflammatory cell death cascades. However, there is a growing body of evidence that links caspase activation to additional cellular functions, independent from those leading to cell death. For instance, *in vitro* and *in vivo* studies indicate that caspase-7 cleaves and is regulated by sterol regulatory element binding proteins (SREBPs), transcription factors bound to endoplasmic reticulum (ER) and nuclear membranes [48], [49]. When cells are depleted of cholesterol, proteolytic cleavage of SREBPs results in their nuclear translocation and subsequent transcription of genes responsible for cholesterol biosynthesis and lipoprotein uptake [50]. Gurcel *et al* demonstrated that activation of SREBPs in response to the pore-forming toxin, aerolysin, promoted intoxicated cell survival in a caspase-1 dependent manner [22]. However, in our studies, activation of caspase-7 did not require caspase-1. Moreover, we observed no analogous upregulation of cholesterol biosynthesis genes (data not shown). Caspases can also regulate virulence determinants during intracellular infection. Caspase-3, a canonical “executioner” caspase highly related to caspase-7, was recently shown to cleave the *Salmonella* type III secreted effector protein SipA [51]. Processing of SipA was necessary for the generation of inflammation in mouse infection models, and inflammatory signaling is critical for *Salmonella* pathogenesis [52], suggesting co-evolution between host and pathogen to promote bacterial virulence in this context. Caspase-mediated cleavage of pathogen-derived proteins can also function to attenuate virulence. For instance, caspase-7 cleavage of ORF57, an early protein critical for viral replication during KSHV infection, inhibits the expression of viral lytic proteins, thereby limiting viral spread [53]. Taken together, these studies demonstrate that caspases play a complex role during microbial infection, and individual caspases may act in a manner that benefits the host and/or the pathogen.

The mechanisms by which the host cell defends itself from LLO-mediated toxicity or damage by other microbial virulence factors are not fully understood, although the concept of plasma membrane or phagosomal membrane damage as a signature of pathogenesis has been recognized [4], [54]. Infection-associated signals, such as flagellin or membrane damage, may be sensed such that weak signals trigger a protective response, whereas strong signals result in initiation of programmed cell death through pyroptosis or apoptosis. Sublytic levels of membrane damage by pore formation may send a “weak” signal, inducing adaptive mechanisms of membrane repair. Indeed, several groups have demonstrated that mammalian cells can tolerate sublytic concentrations of the related cytolysin, Streptolysin O, which causes transient membrane perforations that allows delivery of large macromolecules into live cells [55]–[57]. Repair of such membrane lesions can occur by the exocytosis of lysosomes to the site of injury, followed by endocytosis of the damaged section of membrane [55], [58]. Previous studies indicate that LLO is actively produced by cytosolic *L. monocytogenes* [39] and therefore LLO could cause ongoing transient membrane damage throughout the infection that is kept in check by membrane repair. Investigating the pore forming toxin, BT toxin, from the invertebrate pathogen *Bacillus thuringiensis*, Bischof *et al* reported that intoxication of *C. elegans* and mammalian cells stimulated the unfolded protein response (UPR), a stress-related mechanism aimed at maintaining protein homeostasis in the ER, and that activation of this stress response was critical for nematode defense against the toxin [59]. Whether the UPR is activated in response to LLO toxicity or infection with *L. monocytogenes* is not known, and whether caspase-7 acts analogously or together with the UPR as a defense mechanism to protect host membrane integrity remains to be elucidated. Given that membrane-damaging virulence factors are virtually ubiquitous among bacterial pathogens, we propose that caspase-7 may represent a broad mechanism by which the host cell can sense this common insult and protect itself from ongoing damage.

## Materials and Methods

### Ethics statement

Humane animal care at the University of Michigan is provided by the Unit for Lab Animal Medicine, which is accredited by the American Association for Accreditation of Laboratory Animal Care and the Department of Health and Human Services. This study was carried out in strict accordance with the recommendations in the Guide for the Care and Use of Laboratory Animals of the National Institutes of Health. The protocol was approved by the Committee on the Care and Use of Animals (UCUCA) of the University of Michigan (#A3114-01).

### Bacterial strains and reagents

BMDM were infected with *Listeria monocytogenes* strain 10403S (WT), DP-L2161 (LLO^−^) [60] which has an in-frame deletion of the *hly* gene, DH-616 (iLLO) [40] whose allele of the *hly* gene is controlled by an IPTG inducible promoter, and DPL-1875 [60], a *hly* deletion strain expressing *pfo* (perfringolysin O) using the *hly* promoter. The anti-caspase-7 antibody, which recognizes full length and cleaved caspase-7 (cat #9492) was purchased from Cell Signaling Technology. Rhodamine-phalloidin (cat #R415) was purchased from Molecular Probes. Digitonin and α-hemolysin were purchased from Sigma-Aldrich.

### Cell culture and infection

Bone marrow derived macrophages (BMDM) were isolated from WT C57BL/6 mice, except where targeted mutations were indicated. All genetically deficient strains of mice used were constructed in this background. Briefly, isolated BMDM were differentiated in DMEM with 20% heat-inactivated FBS (Hyclone Laboratories), 1% L-glutamine, 1% sodium pyruvate and 30% L929 fibroblast conditioned medium. Cells were cultured in non-TC treated plates, fed with fresh media on day 3 and harvested on day 6 for infection on day 7. Cultures were maintained in a humidified incubator at 37°C with 5% CO_2_. Overnight cultures of *L. monocytogenes* in BHI broth were incubated statically at 30°C. Prior to infection, bacteria were pelleted and resuspended in PBS.

For experiments with the iLLO strain, cultures were grown overnight statically at 30°C in BHI+1 mM IPTG. Before infection, cultures were back diluted 1∶10 in fresh BHI with 1 mM IPTG and grown with shaking at 37°C until an OD_600_ of 1.2 was achieved, at which point bacteria were pelleted and resuspended in PBS to be used for inoculation of cell cultures. All other strains was treated equivalently, but without IPTG. When iLLO was used to infect cells, 10 mM IPTG was added to the cell culture media for the indicated times post infection. Cells were infected at MOI1 or MOI5 for most strains and MOI10 for iLLO to compensate for decreased phagosomal escape of the iLLO strain. When infected in this manner, the WT and iLLO strains grew with similar kinetics and cells supported equivalent intracellular growth at 4 (data not shown) and 8 h pi (Figure 4B).

### Caspase-7 cleavage

Two million BMDM were plated in 60 mm dishes 18 h prior to infection. Cells were infected at MOI5 for 30 minutes, after which the inoculum was removed, cells washed with PBS and replaced with media containing 10 µg/ml gentamicin. For samples infected at MOI1, cells were spun at 1200 rpm for 3 min to maximize the population of infected cells, after which the inoculum was removed, cells washed, and antibiotic-free media replaced. At 30 min pi, cells were washed again and 10 µg/ml gentamicin was added to inhibit extracellular bacterial replication. At 8 h pi cells were lysed in buffer containing 1% NP-40 on ice for 15 minutes and then spun at 13,000 rpm for 10 min to pellet the insoluble fraction. Soluble fractions were separated by SDS-PAGE, transferred to nitrocellulose membrane, and probed with anti-caspase-7 antibody. Membranes were incubated and treated according to the antibody manufacturer instructions. Membranes from individual gels were cut to optimize exposure times for the cleaved and full-length forms of the protein. Bands were visualized using West Femto chemiluminescent substrate (Thermo Scientific).

### Enzyme activity assays

For DEVDase activity assays, 4 million BMDM were plated in a 96 well format and infected according to the protocol outlined for immunoblotting. At 8 h pi, cells were exposed to the DEVDase substrate per the manufacturer's instructions (Caspase-Glo 3/7 Assay; Promega) and enzyme activity was quantified by luminometer. For LDH assays, BMDM were seeded into 96-well plates at a density of 4 million cells per plate. Prior to infection, medium was replaced with DMEM lacking phenol red. All subsequent steps were performed in this medium. Cells were infected as described above and supernatants harvested at indicated times post infection. Lactate dehydrogenase release was quantified using the Cytox96 Assay Kit (Promega) according to the manufacturer's instruction and quantified by spectrophotometer.

### Intracellular growth assays

*L. monocytogenes* growth curves were performed according to the following protocol. Sterile glass coverslips were placed in 24 wells, onto which 4×10^6^ BMDM were seeded and allowed to adhere overnight. Bacteria were added to the BMDM at MOI1 and allowed to invade for 30 minutes. The inoculum was then removed, the cells were washed three times in PBS, and fresh BMDM media was added with 50 µg/ml gentamicin to inhibit extracellular bacterial growth. For gentamicin washout experiments, the antibiotic was removed from the cultures 2 h pi, and the media was replaced every hour to limit the contribution of extracellular bacteria to intracellular CFU counts. At the indicated time points, coverslips were removed and BMDM were osmotically lysed and serially diluted to enumerate CFU. CFU were counted using the aCOLyte SuperCount (Microbiology International, Fredrick, MD) plate reader and software.

### Immunofluorescence and microscopy

For TUNEL staining, BMDM were plated in 6-well format at a density of 2×10^6^ cells per dish and incubated overnight. The cells were then infected according to the protocol outlined for immunoblotting and stained for DNA fragmentation per the manufacturer's instructions (Roche). For host cell permeability assays, BMDM were seeded onto square coverslips in 6 well plates at a density of 5×10^5^ per well the night before infection. The day of infection, host cells were infected with *L. monocytogenes* for 30 mins, after which the cells were washed 3 times with PBS and cell medium with 10 µg/ml gentamicin was added. Gentamicin was removed from the medium at 2 hpi and cells were washed once per hour with fresh media. At 8 h pi, live cells were washed twice with HBSS+30 mM HEPES, and then stained using 1∶50 rhodamine-phalloidin (Invitrogen) in HBSS+30 mM HEPES for 15 minutes. Coverslips were rinsed using HBSS+30 mM HEPES and fixed in 4% paraformaldehyde. After fixation, coverslips were rinsed three times in TBS+0.1% Triton-X 100 and counterstained with DAPI. Coverslips were mounted onto slides using Prolong Anti-Fade (Invitrogen), and imaged at the Center for Live Cell Imaging (CLCI) at the University of Michigan Medical School using an Olympus BX60 upright fluorescence microscope (Olympus; Center Valley PA). Images were collected using a DP70 CCD color camera (RGB, 12-bits/channel; Olympus America Inc., Center Valley PA) using DP70 controller/manager software v3.02. Automated image analysis was performed using MetaMorph software (Molecular Devices Sunnyvale, CA).

### Listeriolysin O expression, purification, and quantification

For purification of recombinant LLO, 10 mL LB cultures containing 50 µg/ml kanamycin (LB Kan50) sulfate were inoculated with single colonies of *E. coli* BL21(DE3) containing plasmid pET29 that encodes for a 6xHis-tagged copy of the *hly* gene encoding Listeriolysin O (LLO) from *L. monocytogenes* strain 10403S from freshly streaked LB agar plates and incubated overnight at 37°C with constant agitation. Cultures were then used to inoculate 100 ml of LB Kan50 and incubated at 30°C with constant agitation for 30 minutes. IPTG was added to cultures at a final concentration of 1 mM and incubation was resumed for an additional 18 h to induce LLO expression. Protein expression cultures were pelleted at 4000×*g* for 15 min at 4°C and supernatants were discarded. Pellets were resuspended in 1 ml lysis buffer (50 mM sodium phosphate dibasic, 300 mM sodium chloride, 10 mM imidazole; pH 8.0) containing 1 mg/ml lysozyme and incubated on ice for 30 minutes. Suspensions were then subjected to 4×30 sec sonication treatments separated by 15 sec incubation on ice with a Misonix Microson Ultrasonic Cell Disruptor XL set to intensity 4. Lysates were centrifuged for 30 minutes at 16,000×*g* at 4°C. To purify 6xHis-tagged LLO, a NiNTA spin column (Qiagen, Cat. No. 31314) was loaded with 600 µl wash buffer (50 mM sodium phosphate dibasic, 300 mM sodium chloride, 20 mM imidazole; pH 8.0) and centrifuged at 700×*g* for 2 minutes; flow-through was discarded. All subsequent NiNTA spin column centrifugation steps were carried out at 700×*g* for 2 minutes. Lysate supernatants were passed through the column in 600 µL increments. Columns were then washed sequentially with 600 µl volumes of the following: wash buffer, 84% wash buffer/16% glycerol v/v, and wash buffer containing 700 mM NaCl. 6XHis-tagged LLO was then eluted from the column with two 200 µl volumes of wash buffer containing 400 mM imidazole. Combined elution volumes were then passed through an Amicon Ultra 3000 MWCO filter unit by centrifugation for 30 minutes at 16,000×*g* at 4°C, after which 450 µl HBSE (10 mM HEPES, 140 mM NaCl, 1 mM EDTA; pH 8.4) buffer was added to the column, briefly agitated, and collected by flipping the column and centrifuging the solution into a microcentrifuge tube at 16,000×*g* for 10 seconds. Protein solutions were then separated into 50 µl aliquots and stored at −80°C. Final protein concentration was measured by Bradford assay (Thermo Scientific) using a BSA standard curve.

### Statistical analysis

All p values were generated between identified samples using unpaired two-tailed t-tests and represent analysis of ≥3 replicates per condition. * P<0.05, ** P<0.01 and *** P<0.001.

## Supporting Information

Figure S1 **Infection at MOI1 and MOI5 results in equivalent intracellular CFU by 8 h pi.** BL/6 BMDMs were infected at the indicated MOI for 30 min, after which 10 µg/ml gentamicin was added to the cell culture medium to inhibit extracellular bacterial replication. Intracellular CFU were enumerated 8 h pi by osmotic lysis and serial dilution onto LB agar plates.
(PDF)

Figure S2 **Caspase-3 is cleaved in response to** ***L. monocytogenes*** **infection.** BL/6 BMDM were infected with *L. monocytogenes* and cell lysates were probed with an antibody that recognizes full-length and cleaved caspase-3 (Cell Signaling Technology). Cells were treated with 1 µM staurosporine for 4 hrs (stp) as a positive control for caspase-3 cleavage.
(PDF)

Figure S3 **Most** ***L. monocytogenes*** **infected cells do not display common markers of apoptosis.** BL/6 BMDMs were infected at MOI5 with *L. monocytogenes* for 6 h and then stained for phosphotidyl serine exposure using Annexin V according to the manufacturer's instructions (Biotium, Inc. Hayward, CA). The percentage of cells positive for Annexin V was determined via fluorescence microscopy of duplicate samples N>100 cells per condition. Cells were treated with 1 µM staurosporine for 4 hrs (stp) as a positive control for apoptosis. *ns* = not significant by unpaired t-test.
(PDF)

Figure S4 **Caspase-7 deficient cells retain LDH at similar levels as BL/6 cells.** BMDMs isolated from BL/6 and caspase-7-deficient mice were infected at MOI5 with *L. monocytogenes* for 8 h after which release of lactate dehydrogenase (LDH) into cell culture supernatants was determined. The amount of LDH released as the result of detergent induced lysis was set to 100% and the amount of signal from uninfected cells was set to 0%. *ns* = not significant by unpaired t-test.
(PDF)

Figure S5 **WT and iLLO strains grow to equivalent intracellular CFU by 8 h pi.** WT BMDMs were infected using the indicated strains and conditions for 8 h as in Figure S1. Intracellular CFU were enumerated by osmotic lysis of macrophages and serial dilution onto LB agar plates.
(PDF)

Figure S6 **BMDM treated with low concentrations of exogenous LLO do not release LDH by 5 h pi.** BMDM cultures from Figure 7B were washed 1 h post toxin treatment, after the initial LDH release measurement, and fresh medium was added to the cells. LDH released into the medium was measured after 5 h. The amount of LDH released as the result of detergent induced lysis was set to 100% and the amount of signal from uninfected cells was set to 0%.
(PDF)

Figure S7 **BMDM treated with α-hemolysin release little LDH 8 h post treatment.** BMDMs from the experiment shown in Figure 6C and 6D were washed 1 h post toxin treatment, after the initial LDH release measurement, and fresh media was added to the cells. LDH released into the fresh medium was measured 8 h later. The amount of LDH released as the result of detergent induced lysis was set to 100% and the amount of signal from uninfected cells was set to 0%.
(PDF)
