# Supplementary figures and images for: Membrane Damage during Listeria monocytogenes Infection Triggers a Caspase-7 Dependent Cytoprotective Response
Source: PLoS Pathog. 2012 Jul 12;8(7):e1002628. doi: 10.1371/journal.ppat.1002628 (PMC3395620; doi:10.1371/journal.ppat.1002628)

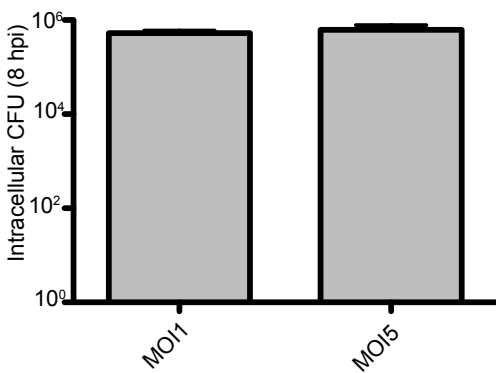

Supplement: Figure S1 — Infection at MOI1 and MOI5 results in equivalent intracellular CFU by 8 h pi. BL/6 BMDMs were infected at the indicated MOI for 30 min, after which 10 µg/ml gentamicin was added to the cell culture medium to inhibit extracellular bacterial replication. Intracellular CFU were enumerated 8 h pi by osmotic lysis and serial dilution onto LB agar plates. (PDF) [file ppat.1002628.s001.pdf]

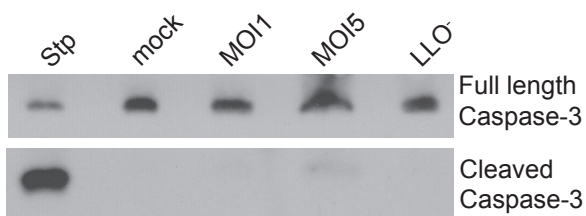

Supplement: Figure S2 — Caspase-3 is cleaved in response to L. monocytogenes infection. BL/6 BMDM were infected with L. monocytogenes and cell lysates were probed with an antibody that recognizes full-length and cleaved caspase-3 (Cell Signaling Technology). Cells were treated with 1 µM staurosporine for 4 hrs (stp) as a positive control for caspase-3 cleavage. (PDF) [file ppat.1002628.s002.pdf]

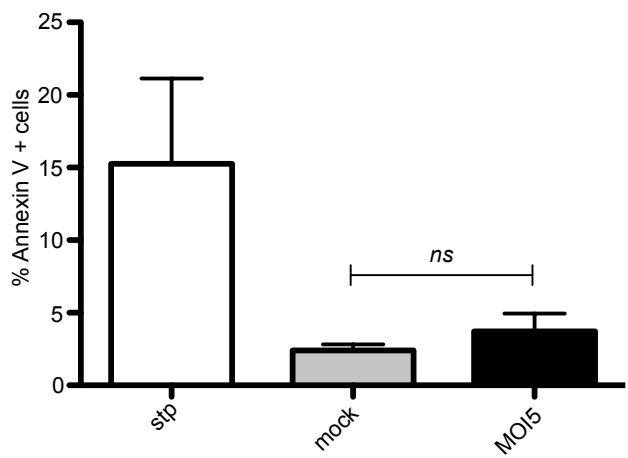

Supplement: Figure S3 — Most L. monocytogenes infected cells do not display common markers of apoptosis. BL/6 BMDMs were infected at MOI5 with L. monocytogenes for 6 h and then stained for phosphotidyl serine exposure using Annexin V according to the manufacturer's instructions (Biotium, Inc. Hayward, CA). The percentage of cells positive for Annexin V was determined via fluorescence microscopy of duplicate samples N>100 cells per condition. Cells were treated with 1 µM staurosporine for 4 hrs (stp) as a positive control for apoptosis. ns = not significant by unpaired t-test. (PDF) [file ppat.1002628.s003.pdf]

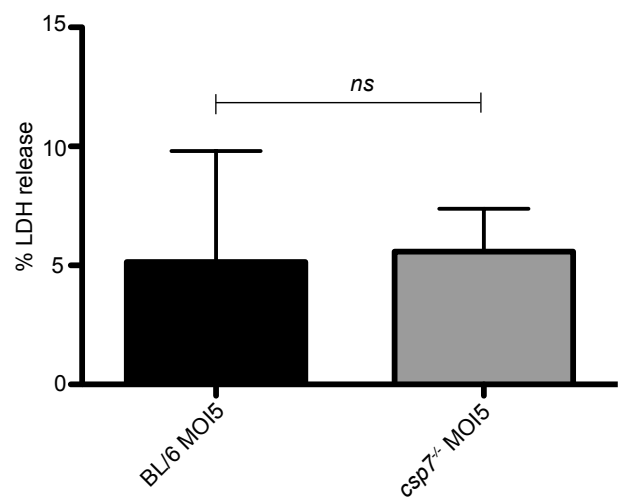

Supplement: Figure S4 — Caspase-7 deficient cells retain LDH at similar levels as BL/6 cells. BMDMs isolated from BL/6 and caspase-7-deficient mice were infected at MOI5 with L. monocytogenes for 8 h after which release of lactate dehydrogenase (LDH) into cell culture supernatants was determined. The amount of LDH released as the result of detergent induced lysis was set to 100% and the amount of signal from uninfected cells was set to 0%. ns = not significant by unpaired t-test. (PDF) [file ppat.1002628.s004.pdf]

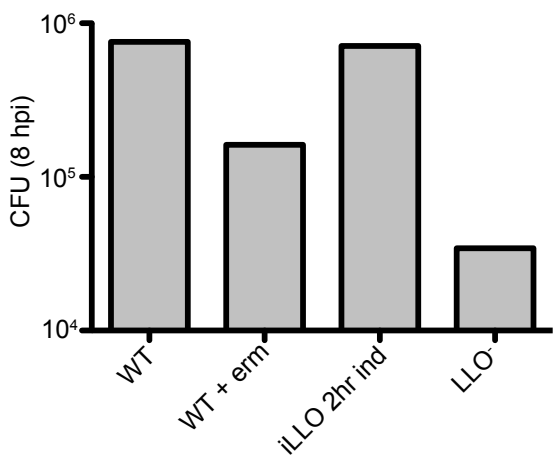

Supplement: Figure S5 — WT and iLLO strains grow to equivalent intracellular CFU by 8 h pi. WT BMDMs were infected using the indicated strains and conditions for 8 h as in Figure S1. Intracellular CFU were enumerated by osmotic lysis of macrophages and serial dilution onto LB agar plates. (PDF) [file ppat.1002628.s005.pdf]

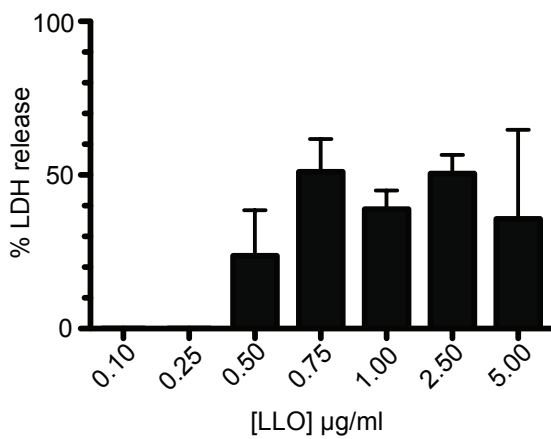

Supplement: Figure S6 — BMDM treated with low concentrations of exogenous LLO do not release LDH by 5 h pi. BMDM cultures from Figure 7B were washed 1 h post toxin treatment, after the initial LDH release measurement, and fresh medium was added to the cells. LDH released into the medium was measured after 5 h. The amount of LDH released as the result of detergent induced lysis was set to 100% and the amount of signal from uninfected cells was set to 0%. (PDF) [file ppat.1002628.s006.pdf]

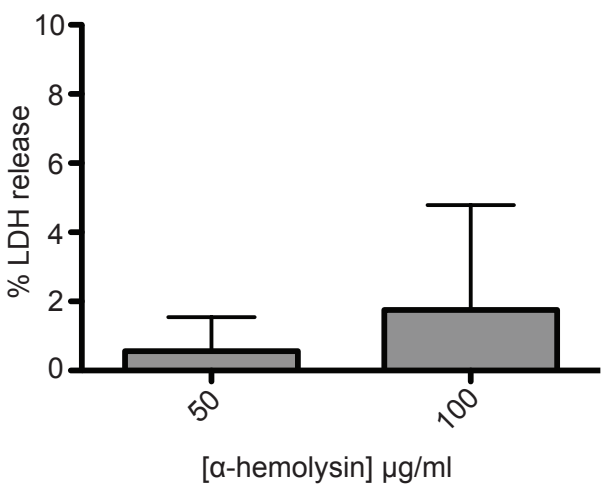

Supplement: Figure S7 — BMDM treated with α-hemolysin release little LDH 8 h post treatment. BMDMs from the experiment shown in Figure 6C and 6D were washed 1 h post toxin treatment, after the initial LDH release measurement, and fresh media was added to the cells. LDH released into the fresh medium was measured 8 h later. The amount of LDH released as the result of detergent induced lysis was set to 100% and the amount of signal from uninfected cells was set to 0%. (PDF) [file ppat.1002628.s007.pdf]
